# Towards a Miniaturized 3D Receiver WPT System for Capsule Endoscopy

**DOI:** 10.3390/mi10080545

**Published:** 2019-08-17

**Authors:** Sadeque Reza Khan, Marc P.Y. Desmulliez

**Affiliations:** Institute of Sensors, Signals and Systems, School of Engineering and Physical Sciences, Heriot-Watt University, Edinburgh EH14 4AS, Scotland, UK

**Keywords:** 3D receiver, capsule endoscopy, phantom, power transfer efficiency, specific absorption rate

## Abstract

The optimization, manufacturing, and performance characterization of a miniaturized 3D receiver (RX)-based wireless power transfer (WPT) system fed by a multi-transmitter (multi-TX) array is presented in this study for applications in capsule endoscopy (CE). The 200 mm outer diameter, 35 μm thick printed spiral TX coils of 2.8 g weight, is manufactured on a flexible substrate to enable bendability and portability of the transmitters by the patients. The 8.9 mm diameter—4.8 mm long, miniaturized 3D RX—includes a 4 mm diameter ferrite road to increase power transfer efficiency (PTE) and is dimensionally compatible for insertion into current endoscopic capsules. The multi-TX is activated using a custom-made high-efficiency dual class-E power amplifier operated in subnominal condition. A resulting link and system PTE of 1% and 0.7%, respectively, inside a phantom tissue is demonstrated for the proposed 3D WPT system. The specific absorption rate (SAR) is simulated using the HFSS^TM^ software (15.0) at 0.66 W/kg at 1 MHz operation frequency, which is below the IEEE guidelines for tissue safety. The maximum variation in temperature was also measured as 1.9 °C for the typical duration of the capsule’s travel in the gastrointestinal tract to demonstrate the patients’ tissues safety.

## 1. Introduction

Capsule endoscopes (CEs) are increasingly used for patients suffering from gastrointestinal (GI) disorders, as the relatively painless procedure allows early stage detection and diagnosis. Since the introduction of the first capsule in the late 1990s [1], increasingly complex systems have been proposed by the scientific community to achieve high-quality diagnostic yields. Subelements for these capsules range from electromechanical actuators for biopsy [2,3], nonwhite-light imaging sensors [4,5], actuators for locomotion [6], to electronic systems [7]. However, the lack of available small size batteries with sufficient capacity for continuous operation prevent their integration into todays CE [8,9]. Therefore, the most promising present alternative is the wireless power transfer (WPT) to the capsule from an external transmitter (TX) [10,11]. 

WPT technology has established itself as a promising candidate for complex medical implantable devices (MIDs), such as neurostimulators, pacemakers, or retinal implants [12,13,14]. Unlike fixed MIDs, endoscopic capsules travel through the gastrointestinal track. Therefore, it is essential to design a method whereby power can be satisfactorily delivered to the capsule irrespective of its location and position with respect to the TX. Moreover, the volume of a typical capsule—11 mm outer diameter by 26 mm length [15]—places severe constraints on the size of the embedded receiver (RX), leading to a low power transfer efficiency (PTE) for the resulting system. 

Different misalignment insensitive RX coils configurations have been reported in the literature for an extensive range of WPT applications. In research of Jonah (et al.) [16] and Liu (et al.) [17], a strongly coupled magnetic resonance-based WPT method is illustrated for 3D power transfer coils whereby two and three orthogonal and circular loops are connected to each other to attenuate the angular misalignment effect. This architecture offers PTE in air of more than 60% at 41.5 MHz [16] and 40% at 80.2 MHz [17], respectively, for the whole range of RX coil angular positions. However, the 100 mm outer diameter of the TX and RX are inappropriate for MIDs, such as CE, due to the strict size restriction of the RX inside the human body. Furthermore, the high operation frequencies of the proposed systems could cause significant tissue heating for long durations of continuous operation as encountered in CE, requiring therefore experimental validation regarding human tissue safety. A 3D WPT system is presented with six and four TX and RX coils, respectively, positioned at different locations of a 46 × 24 × 20 cm^3^ of a home cage of rodent and a box of 11 × 20 × 22 mm^3^ installed on the head of a rat, respectively [18]. At 13.56 MHz operation frequency, PTEs of 23.6–33.3% and 6.7–10.1% have been demonstrated in air for a head height of 8 and 20 cm, respectively. This multiple TX coil-based WPT configuration is, however, exceptionally complex for CE applications. In addition, the proposed 3D RX is significantly (1.5 times) bigger than the typical volume of the capsules leaving therefore no room for sensing modalities. In research of Lenaerts (et al.) [19], a 3D receiver composed of three orthogonal coils stretched inside the 10 mm outer diameter, 13 mm length capsule, achieves a PTE of 1% in air at 1 MHz using a cylindrical TX coil of 41 and 30 cm in diameter and length, respectively, surrounding the patient. The specific absorption rate (SAR) recorded is 0.4 W/kg. In publication of Zhiwei (et al.) [20], a 3D WPT system is proposed for CE, whereby a ferrite slab is used to wind the RX coils around it. The 5% efficiency of the wireless link is measured for a 218 kHz frequency in air, with TX and RX coils of 400 mm and 11.5 mm diameters, respectively. The measured SAR of 8 W/kg is, however, well above the recommended IEEE guidelines regarding tissue safety [21]. A omnidirectional miniaturized RX coil analogous to that in research of Jonah (et al.) [16] and Liu (et al.) [17] is demonstrated in work of Pacini (et al.) [22] for CE WPT applications. However, a 24 × 18 mm^2^ size of the RX is not appropriate for typical CE capsules. A PTE link of 5% in air is, however, achieved for a 7 cm separation distance from a square TX coil of 78 × 52 mm^2^ at 6.78 MHz. In this paper, a 3D RX along with a flexible multi-TX WPT system is proposed. Compared to previously published studies, the proposed architecture offers a miniaturized 3D RX of 8.9 mm diameter and 4.8 mm length, that can be integrated into a typical endoscopic capsule. Furthermore, the proposed WPT system demonstrates a 1% WPT-link efficiency at a 10 cm separation distance and 1 MHz operating frequency inside a muscle phantom. The SAR of 0.66 W/kg confirms tissue safety of the proposed system. The designed flexible and portable TX coils of 200 mm diameter can be easily mounted onto a patient’s body compared to the previously proposed TX coils for CE.

A 3D type RX is therefore proposed in this study to satisfy the requirements of tolerance to capsule misalignment, small size, moderate heat dissipation, and satisfactory PTE. The 3D RX coil is fully investigated using a set of analytical expressions and optimized via a nested multidimensional optimization algorithm presented in research of Khan (et al.) [23]. A multi-transmitter (TX) system is utilized in this work to achieve higher PTE for large TX–RX separation distances. A flexible substrate is used to manufacture the TX coils which can be conformed to the patient’s body and allow easy portability. Tissue safety is simulated in terms of specific absorption rate (SAR) using ANSYS HFSS^TM^ (15.0) and validated experimentally by measuring temperature variations using a tissue phantom.

## 2. Full Characterization of the 3D RX Coils System 

**Figure 1:**
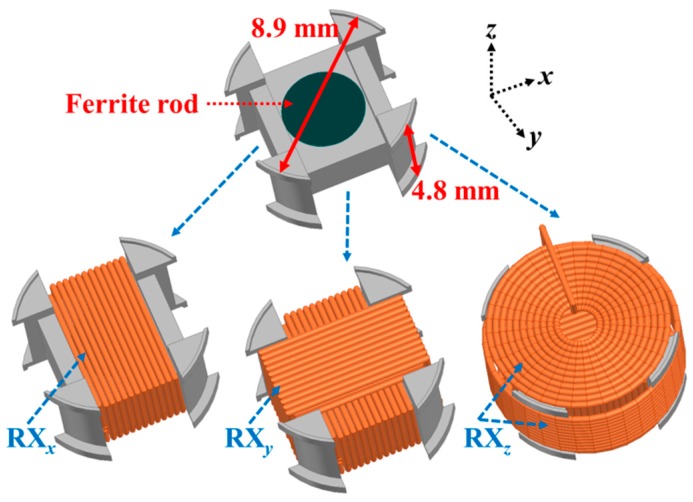
Coil holder architecture representation of the proposed 3D receiver (RX) coil.

Figure 1 shows the proposed 3D RX coils system with and without copper wires. The radius *R_F_* and height *h_F_* of a ferrite rod are included in the system’s core to increase the magnetic flux developed within the space enclosed by the coils allowing thereby the enhancement of TX and RX coils’ mutual coupling [24]. The diameter and thickness of the holder are 8.9 and 4.8 mm, respectively. The three coils are defined as RX*_x_*, RX*_y_*, and RX*_z_*. Each coil, of wire diameter *w*, is assumed to have *N_l_* loops and *N_t_* layers.

### 2.1. Derivation of the Self-Inductance of the 3D RX Coil’s Structure

The self-inductance, *L*_self_, of each WPT coil (RX and TX) can be estimated as [25]:

(1) $$L_{\mathrm{self}}=\mu \overset{N_{l}}{\underset{i=1}{\sum }}R_{i}\left[\ln\left(\frac{16R_{i}}{w}\right)-2\right]+2\mu \overset{N_{l}}{\underset{i=1}{\sum }}\overset{N_{l}}{\underset{j=1}{\sum }}\frac{\sqrt{R_{i}R_{j}}}{\alpha_{ij}}\left[\left(1-\frac{\alpha_{ij}^{2}}{2}\right)K\left(\alpha_{ij}\right)-E\left(\alpha_{ij}\right)\right]\times \left(1-\delta_{ij}\right)\;+2\mu \overset{\left(N_{t}-1\right)}{\underset{i=1}{\sum }}\overset{N_{l}}{\underset{j=1}{\sum }}\overset{N_{l}\times \left(N_{t}-i\right)}{\underset{k=1}{\sum }}\frac{\sqrt{R_{j}R_{k}}}{\alpha_{ij}}\left[\left(1-\frac{\alpha_{jk}^{2}}{2}\right)K\left(\alpha_{jk}\right)-E\left(\alpha_{jk}\right)\right]$$

with

(2) $$\alpha_{ij}=2\sqrt{\frac{R_{i}R_{j}}{{\left(R_{i}+R_{j}\right)}^{2}+d_{ij}{}^{2}}}$$

where *δ_ij_* = 1 *for i* = *j; δ_ij_* = 0*,* otherwise. Furthermore, *μ* is defined as the medium permeability and *d_ij_* is the distance between two layers of loops radii *R_i_* and *R_j_*. The second term of Equation (1) refers to the case of different loops in the same layer, whereas, in the third term, *R_k_* is the radius of a loop in different perfectly aligned layers. In Equation (1), the complete elliptic integrals of the first and second kind are presented as *K* and *E*, respectively. The coils are assumed to be wound as Archimedean spirals [25]. Considering the ferrite effect *L*_self_ is rewritten as [24]:

(3) $$L_{F}=L_{\mathrm{self}}\left[2-\frac{R_{F}^{2}}{R^{2}}\left(1-\frac{\mu_{F}}{1+D_{c}\left(\mu_{F}-1\right)}\right)\right]$$

where *R*, *D_c_*, and *μ_F_* are the outer radius of the coil, demagnetizing factor, and relative intrinsic permeability of the ferrite material, respectively. *D_c_* is defined as [24]:

(4) $$D_{c}=\frac{3.04\zeta^{-0.056}R_{F}^{2}}{h_{F}^{2}e^{3}}\left[e-\tan^{-1}e\right],\;e=\sqrt{1-\zeta^{2}}$$

where *ζ* = *h_F_*/2*R_F_*. The expression for *D*_c_ is only applicable for *ζ* < 1.

**Figure 2:**
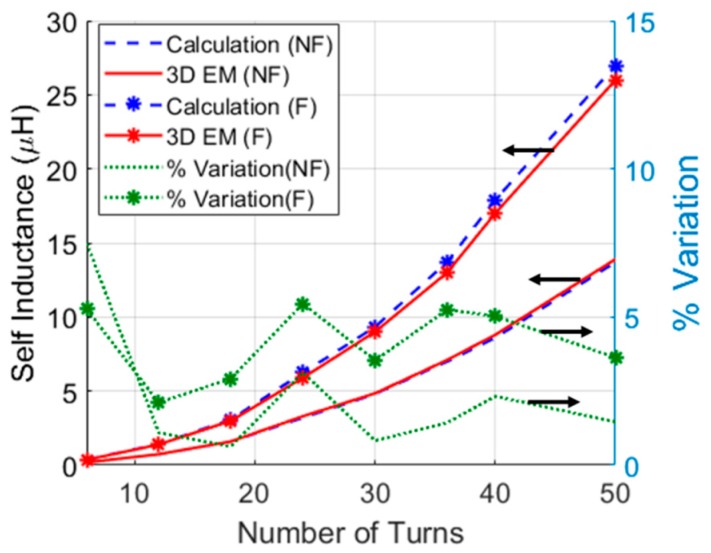
Self-inductance as a function of total number of turns, *N_l_* × *N_t_*, without (NF) and with ferrite (F). The % variation between the analytical and 3D electromagnetic (EM) simulated results is illustrated on the vertical right axis. *R_F_* = 2.5 mm, *h_F_* = 2 mm, and *w* = 0.3 mm.

Figure 2 demonstrates the calculated and simulated self-inductance values as a function of the total number of turns, *N_l_* × *N_t_*, without (WOF) and with ferrite (WF), respectively. Simulations were carried out using the 3D electromagnetic (EM) solver ANSYS HFSS^TM^. The maximum relative difference between the calculated and simulated results in the ferrite case was only 6% for *R_F_* = 2.5 mm, *h_F_* = 2 mm, and *w* = 0.3 mm. Furthermore, the frequency of operation, *f*, was selected as 1 MHz. This frequency was chosen for tissue safety, it also offers cheaper electronic circuitry compared the ISM band of 6.78 MHz in order to demonstrate the concept presented here. It must be noted that the coil’s *R* varies with respect to the number of *N_l_*.

### 2.2. Derivation of the Mutual Inductance of the 3D RX Coil’s Structure

The mutual inductance, *M*, of two current carrying loops, defined as C1 and C2 of respective radii *R_C_*_1_ and *R_C_*_2_ is proposed in research of Khan (et al.) [25] as:

(5) $$M\left(R_{C1},R_{C2},d_{r},\theta ,\lambda \right)=\frac{\mu \pi R_{C1}^{2}R_{C2}^{2}\cos\theta \cos\lambda }{2{\left(R_{C1}^{2}+R_{C2}^{2}+d_{r}^{2}+d_{2}^{2}\right)}^{\frac{3}{2}}}\;\times \left[\begin{array}{c} 1+\frac{15}{64}\left(\gamma_{a}^{2\;}+4\gamma_{b}^{2\;}\right)+\frac{15}{64}\left(\gamma_{a}^{2\;}+4\gamma_{c}^{2\;}\right)\\ \cos^{2}\theta \cos^{2}\lambda +\frac{15}{8}\gamma_{d}^{2\;}\left(\sin\theta \cos\lambda \sin\lambda \cos\theta \right)\\ -\frac{15}{8}\gamma_{c}\gamma_{d}\cos\theta \cos\lambda \left(\sin\theta \cos\lambda +\sin\lambda \cos\theta \right)\\ -\frac{3}{2}\left(\delta_{a}-\delta_{b}\left(\tan\theta +\tan\lambda \right)\right) \end{array}\right].\;$$

For a separation distance, *d_r_*; translational distance, *d*_2_; roll and pitch rotational angle, *θ* and *λ*, respectively. The parameters *γ_a_*, *γ_b_*, *γ_c_*, *γ_d_*, and *γ_e_* are lesser than unity and explained in research of Khan (et al.) [25], together with *δ_a_* and *δ_b_*. Finally, the total mutual inductance, *M*_total_, for *T_C_*_1_ layers and *L_C_*_1_ loops for coil C1 and *T_C_*_2_ layer and *L_C_*_2_ loops for coil C2 is written as [24,25]:

(6) $$M_{\mathrm{total}}=\;\overset{T_{C1}}{\underset{i=1}{\sum }}\overset{T_{C2}}{\underset{j=1}{\sum }}\overset{L_{C1}}{\underset{k=1}{\sum }}\overset{L_{C2}}{\underset{l=1}{\sum }}M\left(\begin{array}{c} R_{C1:k},R_{C2:l},\\ d_{r\left(i,j\right)},d_{2},\theta ,\lambda \end{array}\right)\times \left[2-\frac{R_{F}^{2}}{R^{2}}\left(1-\frac{\mu_{F}}{1+D_{c}\left(\mu_{F}-1\right)}\right)\right]$$

where *R_C_*_1:*k*_ and *R_C_*_1*:l*_ are the radii of the *k*^th^ loop of C1 and *l*^th^ loop of C2, respectively. Furthermore, *d_r_* (*i,j*) is defined as [25]:

(7) $$d_{r}=d_{r\left(N_{t,C1},N_{t,C2}\right)}=d_{1}+\left[\left(N_{t,C1}-1\right)\times \left(w_{C1}+s_{t,C1}\right)\right]+\left[\left(N_{t,C2}-1\right)\times \left(w_{C2}+s_{t,C2}\right)\right]$$

where *d*_1_ is the top and bottom layer’s center-to-center distance of C1 and C2, respectively. Wire diameter and spacing between loops of a coil *C_i_* are defined as *w_Ci_* and *s_t,Ci_*, respectively.

### 2.3. AC Resistance of the WPT Coil

The DC resistance, *R_DC_*, is the significant part of the AC resistance of a WPT coil and calculated as [23,26,27]:

(8) $$R_{DC}=\frac{8R\rho N_{t}l}{w^{2}}$$

where *ρ* is the copper wire resistivity and *l* is the length of the loops in one layer as defined in research of Khan (et al.) [23]. The AC resistance, *R_AC_*, is the addition of skin and proximity effect resistors, *R*_skin_ and *R*_prox_, respectively, and can be written as [28]:

(9) $$R_{AC}=R_{\mathrm{skin}}+R_{\mathrm{prox}}$$

*R*_skin_ can be asymptotically represented as [29]:

(10) $$R_{\mathrm{skin}}=\frac{R_{DC}w^{2}\;}{4\delta \left(w-\delta \right)}$$

where *δ* is the skin depth [16,17,18]. In addition, *R*_prox_, is defined as [30,31]:

(11) $$R_{\mathrm{prox}}=\;\frac{w^{4}}{32}\pi^{3}{\left(\alpha \omega \mu \right)}^{2}\sigma_{c}RlN_{t}$$

where *σ_c_* is the conductivity of the copper and *ω* = 2*πf* is the radial operation frequency. The shape factor, *α*, is calculated as [30]:

(12) $$\alpha =\sqrt{\frac{\sum_{i=1}^{N_{l}\times N_{t}}R_{i}\times H_{i}^{2}}{\sum_{i=1}^{N_{l}\times N_{t}}R_{i}\times I_{i}^{2}}}$$

where *H_i_* and *I_i_* are the intensity of the applied magnetic field and current flowing through to the *i*^th^ wire, respectively [32].

The simplified closed form alternative current (AC) resistance with the effect of ferrite on the copper wire in the 3D RX is provided in publication of Theilmann (et al.) [24], and estimated using the iterative EM simulation as:

(13) $$R_{F}=R_{\mathrm{AC}}\times \frac{\zeta R}{0.001\zeta^{-0.056}}$$

**Figure 3:**
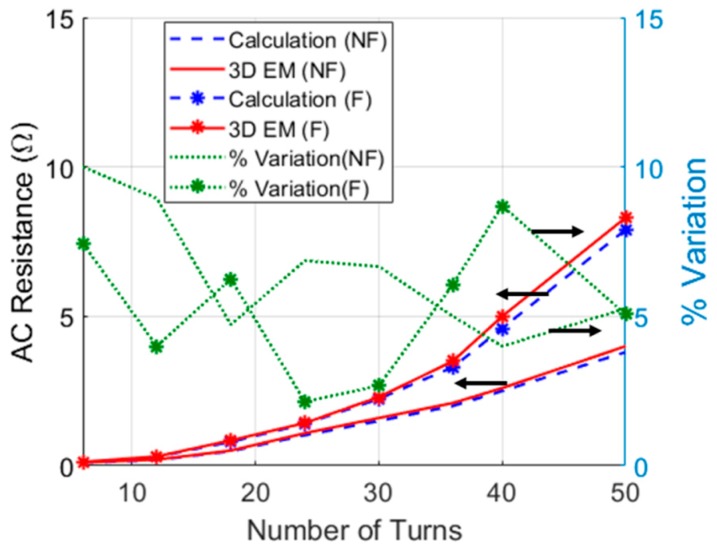
AC resistance presented as a function of total number of turns, *N_l_* × *N_t_*, without (NF) and with ferrite (F). The % variation between calculation and 3D EM simulation is illustrated on the vertical right axis.

Taking the expression of *R_AC_* of the printed spiral coil (PSC) in research of Khan (et al.) [23], and the same coils and ferrite dimensions, Figure 3 shows the analytical and 3D EM simulated AC resistance without (WOF) and with ferrite (WF) as a function of the total number of turns at *f* = 1 MHz. 

The maximum percentage of variation between the calculated and simulated results was less than 10%. The higher % variation at a lower number of turns is due to the very small value of resistance. The parasitic capacitance influence analysis of the copper wire and PSC are explained in research of Yang (et al.) [33] and Khan (et al.) [23], respectively.

## 3. Optimization of the Multi-TX and 3D RX System 

**Figure 4:**
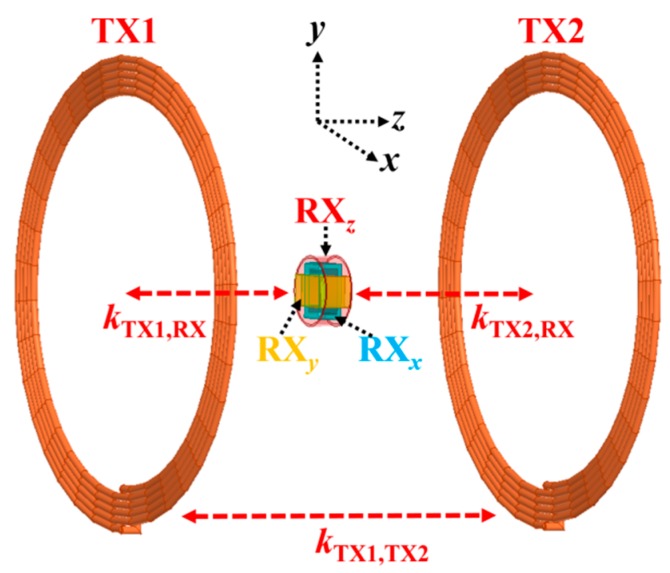
Concept diagram of the multi-transmitter (multi-TX) and 3D RX wireless power transfer (WPT) system.

A concept diagram of multi-TX and 3D RX WPT systems is presented in Figure 4, where *k_i,j_* = ($M_{ij}/\sqrt{L_{i}L_{j}}$) is the coupling coefficient between coils *C_i_* and *C_j_* [11,26,27]. The TX coils are externally powered with the driving sources *F*_1_(*t*) and *F*_2_(*t*). In this case, only the *z*-axis of the 3D RX coil is considered. The RX coil output is associated with the load resistance, *R_L_*. Moreover, the coupling rate between coils *C_i_* and *C_j_* is defined as *K_ij_* (≈*K_ji_*). The multi-TX system can be illustrated by using the coupled-mode theory as [34,35]:

(14) $$\Gamma_{\mathrm{TX}1}A_{\mathrm{TX}1}-jK_{\mathrm{TX}1,\mathrm{TX}2}A_{\mathrm{TX}2}-jK_{\mathrm{TX}1,\mathrm{RX}}A_{\mathrm{RX}}=0$$

(15) $$\Gamma_{\mathrm{TX}2}A_{\mathrm{TX}2}-jK_{\mathrm{TX}2,\mathrm{TX}1}A_{\mathrm{TX}1}-jK_{\mathrm{TX}2,\mathrm{RX}}A_{\mathrm{RX}}=0$$

(16) $$\left(\Gamma_{\mathrm{RX}}+\Gamma_{L}\right)A_{\mathrm{RX}}-jK_{\mathrm{RX},\mathrm{TX}1}A_{\mathrm{TX}1}-jK_{\mathrm{RX},\mathrm{TX}2}A_{\mathrm{TX}2}=0$$

where *Γ_i_* is the resonance width or rate of intrinsic decay of transmitter TX*i*, receiver RX, or load *L*, due to the object absorption and radiation losses. The field amplitude inside a coil is *a_i_*·(*t*) = *A_i_*·*e*^−*jωt*^. Considering *K*_TX1,TX2_ to be negligible due to the large distance between TX1 and TX2, *A*_TX1_/*A*_RX_ and *A*_TX2_/*A*_RX_ can then be written as:

(17) $$\frac{A_{\mathrm{TX}1}}{A_{\mathrm{RX}}}=\frac{K_{\mathrm{TX}1,\mathrm{RX}}\left(\Gamma_{\mathrm{RX}}+\Gamma_{L}\right)\Gamma_{\mathrm{TX}2}}{\left(\begin{array}{c} jK_{\mathrm{TX}1,\mathrm{RX}}^{2}\Gamma_{\mathrm{TX}2}+jK_{\mathrm{TX}2,\mathrm{RX}}^{2}\Gamma_{\mathrm{TX}1} \end{array}\right)}$$

(18) $$\frac{A_{\mathrm{TX}2}}{A_{\mathrm{RX}}}=\frac{K_{\mathrm{TX}2,\mathrm{RX}}\left(\Gamma_{\mathrm{RX}}+\Gamma_{L}\right)\Gamma_{\mathrm{TX}1}}{\left(\begin{array}{c} jK_{\mathrm{TX}1,\mathrm{RX}}^{2}\Gamma_{\mathrm{TX}2}+jK_{\mathrm{TX}2,\mathrm{RX}}^{2}\Gamma_{\mathrm{TX}1} \end{array}\right)}$$

The power at coil *i* can be defined as *P_i_* = 2*Γ_i_*·|*A_i_*|^2^. The total power delivered to the system from the source is *P*_TOT_ = *P*_TX1_ + *P*_TX2_ + *P*_RX_ + *P*_L_. The power transfer efficiency, PTE, of the system, *η* defined as the ratio between the power delivered to the load and the total power delivered, is therefore:

(19) $$\eta =\frac{P_{L}}{P_{\mathrm{TOT}}}=\frac{1}{2+\frac{\Gamma_{\mathrm{RX}}}{\Gamma_{L}}\left[2+\frac{\Gamma_{\mathrm{TX}1}}{\Gamma_{\mathrm{RX}}}{\left|\frac{A_{\mathrm{TX}1}}{A_{\mathrm{RX}}}\right|}^{2}+\frac{\Gamma_{\mathrm{TX}2}}{\Gamma_{\mathrm{RX}}}{\left|\frac{A_{\mathrm{TX}2}}{A_{\mathrm{RX}}}\right|}^{2}\right]}$$

or

(20) $$\eta =\frac{1}{2+\frac{Q_{L}}{Q_{\mathrm{RX}}}\left[2+\frac{1}{{\mathrm{FOMD}}^{2}}{\left(1+\frac{Q_{\mathrm{RX}}}{Q_{L}}\right)}^{2}\right]}$$

where *Q*_RX_ and *Q_L_* (= 2*πf*·*L*_RX_/*R_L_*) are the quality factor of the RX coil and load, respectively. *L*_RX_ is the inductance of the coils. The distance dependent figure of merit (FOMD) is defined as:

(21) $$\mathrm{FOMD}=\sqrt{k_{\mathrm{TX}1,\mathrm{RX}}^{2}Q_{\mathrm{TX}1}Q_{\mathrm{RX}}+k_{\mathrm{TX}2,\mathrm{RX}}^{2}Q_{\mathrm{TX}2}Q_{\mathrm{RX}}}$$

where *Q*_TX1_ and *Q*_TX2_ are the Q-factors of the TX1 and TX2 coils. Expression for the optimized load, $R_{L}^{*}$ is:

(22) $$R_{L}^{*}=\frac{2\pi fL_{\mathrm{RX}}\sqrt{1+2{\mathrm{FOM}}^{2}}}{Q_{\mathrm{RX}}}$$

Total PTE for the two TX and RX 3D WPT systems is then defined as:

(23) $$\eta_{\mathrm{total}}=\eta_{\mathrm{RX}}\left(x\text{-}\mathrm{axis}\right)+\eta_{\mathrm{RX}}\left(y\text{-}\mathrm{axis}\right)+\eta_{\mathrm{RX}}\left(z\text{-}\mathrm{axis}\right)$$

A nested multi-dimensional optimization algorithm presented in research of Khan (et al.) [23] is adopted to optimize the proposed multi-TX and 3D RX WPT systems using Equations (1)–(23). This algorithm extracts coil parameters such as wire width and number of turns to achieve maximum WL-PTE. The complete optimization routine requires approximately 20 min for the Intel (R) Xeon (R) CPU E5-2640 with processor speed of 2.5 GHz and 128 GB of RAM (HP Z820 workstation). The optimized load, $R_{L}^{*}$, is calculated as 6.5 Ω for *d_r_* = 10 cm. The optimized coil parameters of TX and 3D RX are listed in Table 1, where *s* and *N* are the spacing between two copper wires and total number of turns, respectively.

## 4. Experimental Setup 

**Figure 5:**
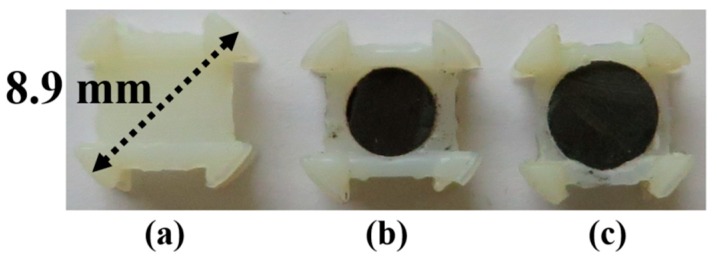
Manufactured coil holders. (**a**) Without ferrite. (**b**) With 4 mm diameter ferrite. (**c**) With 5 mm diameter ferrite.

3D printed coil holders without and with a ferrite rod are proposed as 3D RX demonstrators (Figure 5). 3D printing of the coil holder was carried out using Vero white material produced by Stratasys (Eden Prairie, Minnesota, United States) and an Object500 Connex printer. The outer diameter of the coil holder without the ferrite, Figure 5a, is 8.9 mm. The material 61 Nickel Zinc (NiZn) ferrite rod of height *h_F_* = 2 mm and radius *R_F_* = 2 and 2.5 mm, shown in Figure 5b,c, respectively, was adopted to improve the PTE of the 3D WPT system for the same outer diameter of the holder. The outer diameter and thickness of the proposed 3D RX and its copper wire winding were 8.9 and 4.8 mm, respectively. 

**Figure 6:**
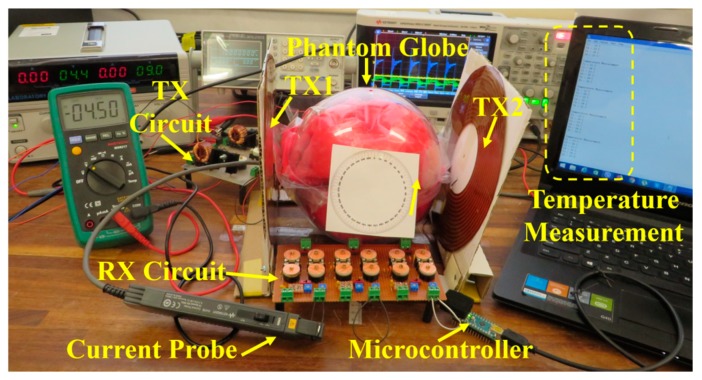
Full experimental setup.

The complete experimental setup with the multi-TX coils and 18 cm diameter spherical phantom is presented in Figure 6. The optimized parameters presented in Table 1 were used to fabricate the TX and RX coils. The TX coils are manufactured on a DuPont^TM^ Pyralux AP flexible substrate of 150 µm thickness with a 35 µm thick copper cladding. The TX coils were placed 1 cm away from the outer surface of the phantom globe, resulting in a total distance of 20 cm between the RX. The TX coils were driven by separate TX circuits (class-E amplifier) fabricated on a single circuit board labeled TX circuit in Figure 6. The phantom with similar electrical properties as human muscle tissue was prepared using hydrophilic organic powder and degassed water [7]. A RX circuit test bench with variable capacitors to adjust resonant conditions of the RX coils and optimize load resistors was used to measure the total WL-PTE as defined in Equation (23). The Schottky diode-based bridge rectifier for each RX coil was also embedded in the RX circuit to measure the complete system efficiency. PTE was measured using a Keysight technologies current probe (1147B) and oscilloscope. The Q-factors of the TX and RX coils at different frequencies were measured using the impedance analyzer HP 4192A. Temperature variation inside the phantom globe was measured by a Microcontroller Arduino Nano 3.0, using a serial communication window.

**Figure 7:**
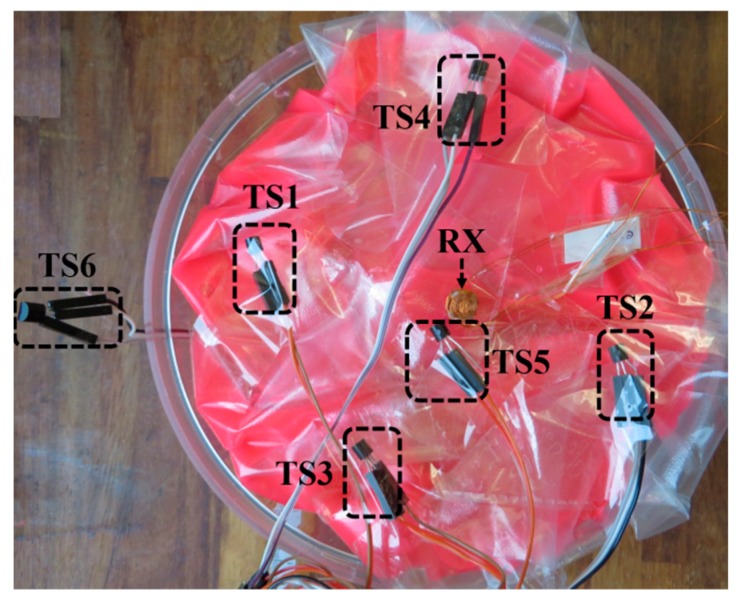
3D RX coil and temperature sensor installation inside the phantom.

Figure 7 shows the cross-section of the phantom globe, which is made of two identical hemispheres filled with the phantom tissues described above. The 3D RX coil was mounted at the center, and five temperature sensors (TSs) were inserted approximately equidistantly around the RX coil. A sixth thermistor, TS6, was placed outside the phantom to measure room temperature.

## 5. Results and Discussion 

**Figure 8:**
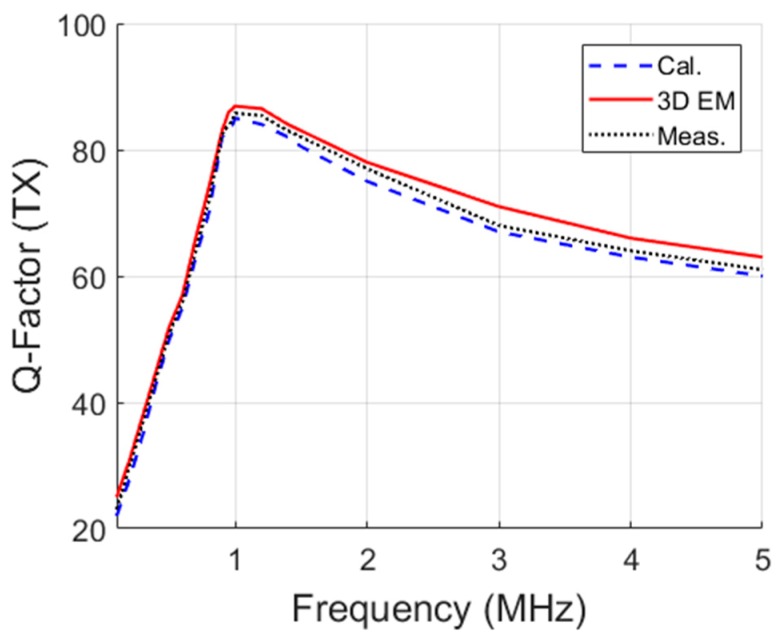
TX coil Q-factor with frequency.

**Figure 9:**
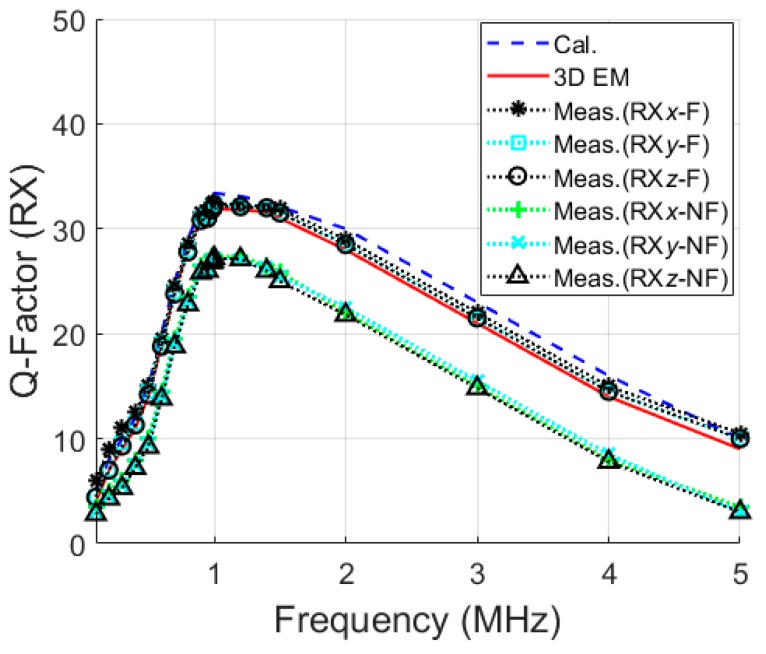
RX coil Q-factor with frequency.

Variations in frequency of the theoretical, simulated, and measured values of the Q-factors for TX and RX coils are presented in Figure 8 and Figure 9, respectively. For the RX coil, the three axes are shown for the frequency range of 500 kHz to 5 MHz. For each frequency, the ideal load, R^*^_L_ was calculated. Excellent agreement was recorded between calculated, simulated, and measured values for both figures.

Variations in Q-factors for RX coils with (F) and without (NF) ferrite rods are displayed in Figure 9. The highest *Q*-factor was achieved at around 1 MHz.

**Figure 10:**
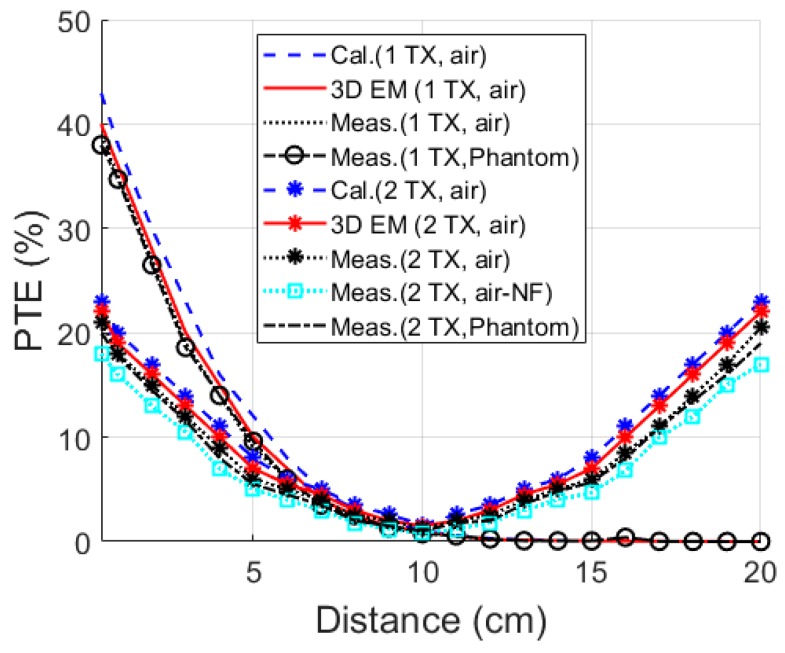
Power transfer efficiency (PTE) of the link as a function of separation distance between TX and RX at 1 MHz operation frequency.

PTE variations are shown in Figure 10 for a separation distance between TX and RX ranging from 0.5 to 20 cm. PTEs for both single and multi-TX coils are recorded in air and phantom. A high PTE was recorded for a single-TX coil at a small distance compared to multi-TX. A significant drop in the PTE after 8 cm was noticed with PTE becoming negligible at 20 cm. In contrast, the multi-TX coils demonstrate promising PTE in the 5–15 cm distance, which is the location of the GI tract in an average human body. The measured PTEs for the 10 cm distance of 3D RX with ferrite rod (*R_F_* = 2 mm) from a single TX coil in air and phantom were 0.9 and 0.75%, respectively. Increased PTE at 1.3 and 1% was achieved for a multi-TX WPT system in air and phantom, respectively. No significant difference was observed in air or in the phantom due to the low absorption of the phantom tissue at 1 MHz. Furthermore, the measured PTE for the 3D RX without ferrite rod (NF) and multi-TX was 0.8% at 10 cm distance in air. A similar WPTE was achieved for 3D RX with *R_F_* = 2.5 mm and the multi-TX WPT system.

**Figure 11:**
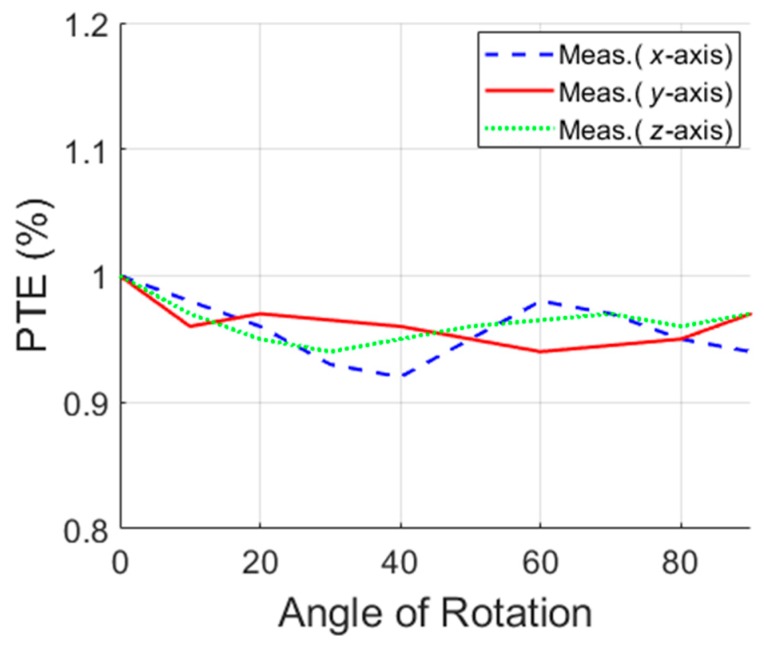
PTE versus angular rotation in different axis.

**Figure 12:**
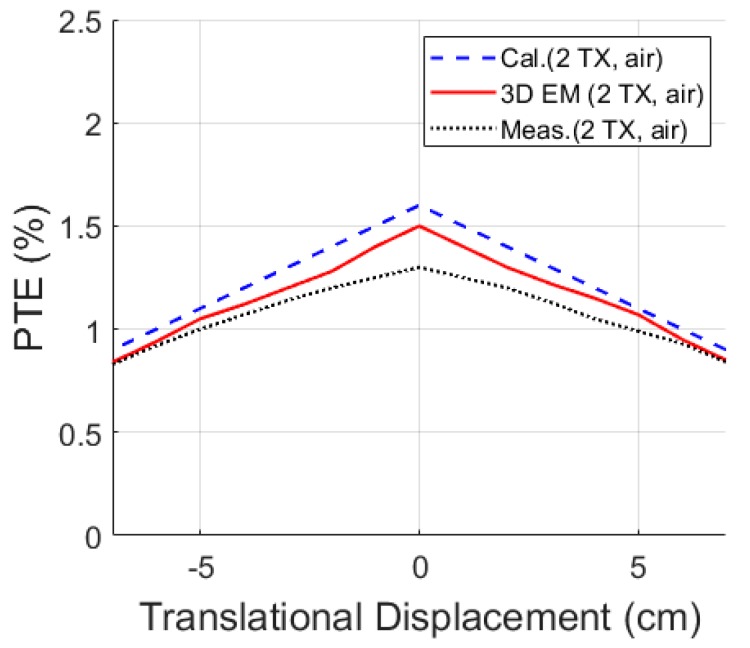
PTE versus translational displacement (*d_r_* = 10 cm).

The total measured PTE as shown in Equation (23), is shown in Figure 11 for angular rotation of 0° to 90° for the phantom globe in the *x*-, *y*-, and *z*-axes. The WPT system maintains total PTE over 0.9% for complete rotation of the 3D RX inside the phantom validating, thereby the significant robustness of the proposed WPT system against the angular movement of the capsule inside the GI tract. The translational misalignment from −7 cm (left) to +7 cm (right), shown in Figure 12, indicates a minimum measured WL-PTE of 0.84% in air and 0.7% in the phantom.

**Figure 13:**
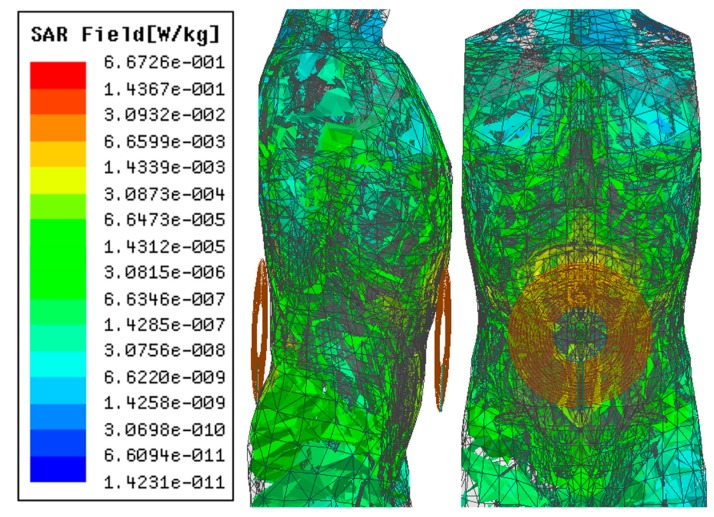
Specific absorption rate (SAR) simulation in a human model in ANSYS HFSS (*f* = 1 MHz).

**Figure 14:**
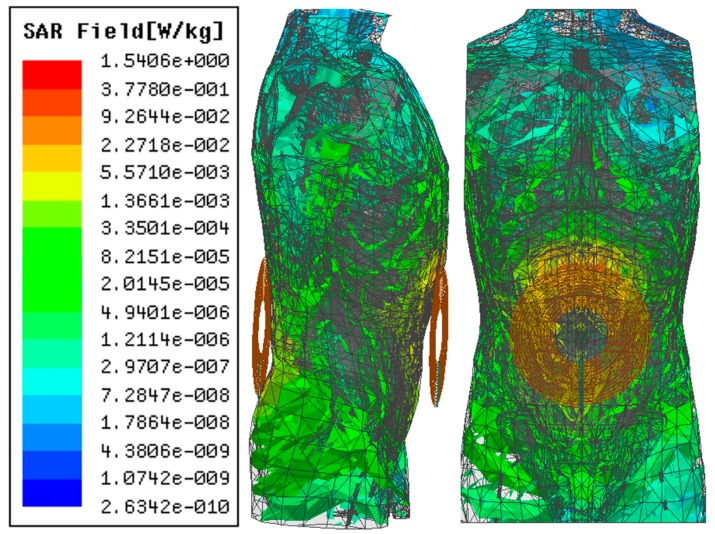
SAR simulation in a human model in ANSYS HFSS (*f* = 6.78 MHz).

SAR simulation results using the ANSYS HFSS^TM^ human model at 1 MHz and 6.78 MHz (ISM band) are shown in Figure 13 and Figure 14, respectively, for 10 W of input power. The SAR obtained at 1 MHz, 0.66 W/kg, is lower than the IEEE standard of 2 W/kg for 10 g of tissue [21]. However, the SAR was recorded at 1.54 W/kg for 6.78 MHz of operation frequency, which is significantly closer to the IEEE standard of tissue safety. 

**Figure 15:**
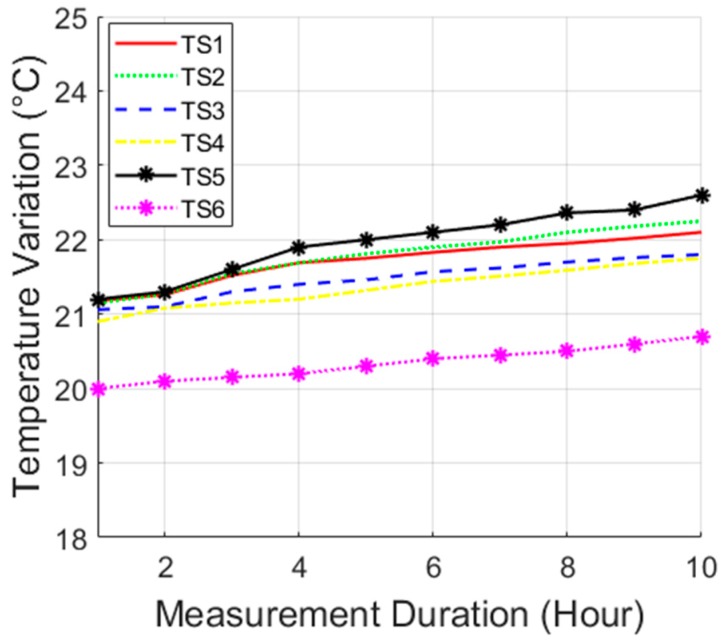
Variation of temperature in the complete capsule endoscopy (CE) duration.

Temperature rise inside the tissue was also measured in the phantom using multiple TSs for a typical capsule transit time of 10-hour inside the GI tract, shown as Figure 15.

The maximum temperature variation in the body (TS5) was 1.9 °C for a variation of 1.4 °C of room temperature (TS6). Temperature rise measurement in and around the capsule should also account for the additional temperature rise generated by the electronic processing inside the capsule to implement the requested sensing modalities as reported in research of Faerber (et al.) [7] and Lay (et al.) [36].

The figure of merit, *FOM*,

(24) $$\mathrm{FOM}=\frac{\mathrm{PTE}\times \mathrm{separation}\;\mathrm{distance}\times \mathrm{axes}}{\mathrm{TX}\;\mathrm{diameter}\times \mathrm{RX}\;\mathrm{size}\times f\times {\mathrm{SAR}}^{2}}$$

proposed in research of Khan (et al.) [37], is adopted here to compare the performance of the various WPT systems for CE with the proposed work. A higher FOM represents better WPT performance. In Equation (24), the term “axes” represents the many directions where the RX can receive power, which is three in our work. Results are presented in Table 2. 

In research of Zhiwei (et al.) [20], the proposed 3D WPT system achieved PTE of ~5% in air at 218 kHz frequency for a nonportable TX coil and higher SAR than recommended by the IEEE guidelines [21]. The WPT systems presented in research of Na (et al.) [38] and Ding (et al.) [39] are either sensitive to misalignment or display a high SAR [39]. The recent 3D WPT system presented in publication of Khan (et al.) [37] demonstrates promising PTE and SAR for CE application. The RX size (8 × 8 mm^2^) is appropriate for typical CE. In this study, the proposed 3D RX coil is, however, smaller in size compared to previous works and offers therefore more space for other electronic processing circuitry or sensing modalities. However, PTE is lower compared to some previous works.

**Figure 16:**
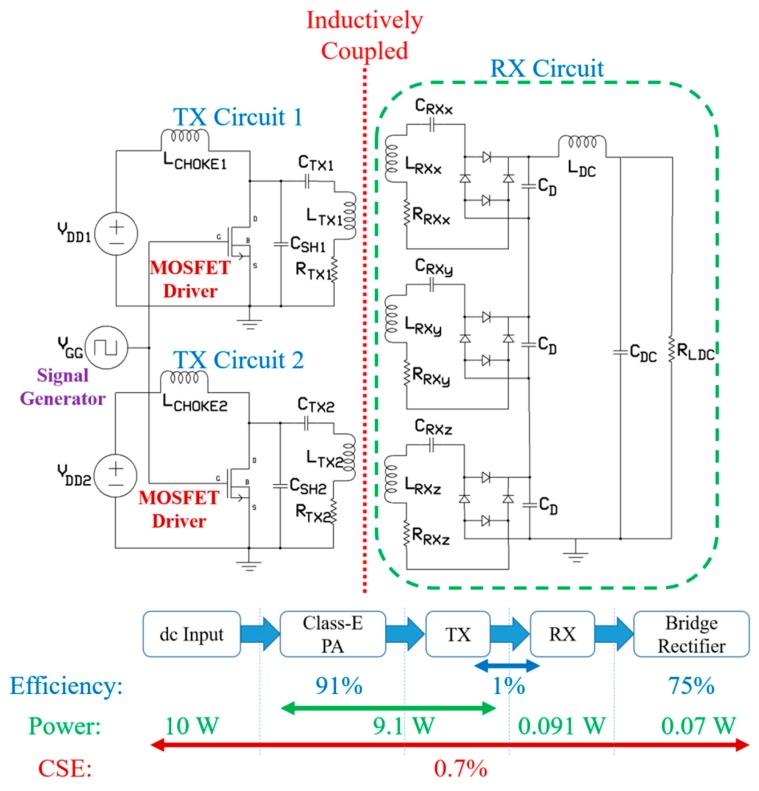
Efficiency of the complete WPT system and efficiency analysis.

The complete WPT system with its electronic processing is presented in Figure 16, alongside analysis of the complete system efficiency (CSE). Two separate TX circuits were used to supply power to the multi-TX WPT system using a single signal generator. It is necessary to use same signal generator for both the TX coils to maintain the excitation with signals having the same phase. Phase variation would otherwise reduce drastically the PTE of the proposed system. A DC-fed energy injection single-ended class-E power amplifier (PA) was adopted for the unit TX circuit [40]. The closed-form equations of the subnominal model presented in research of Liu (et al.) [41] was utilized to design the PA at 1 MHz operating frequency. The IRF640 N-channel metal oxide field effect transistor (MOSFET) and EL7457 noninverting MOSFET driver in between signal generator and MOSFET were used to implement the PA.

Adjustable capacitors were used as shunt capacitors, *C*_SH_ = 7 nF, and series resonant capacitors, *C*_TX_ = 894 pF, for both TX circuits. The parasitic capacitance (400 pF according to the datasheet) of the MOSFET was also included in *C*_SH_. A radio frequency choke (RFC) inductor, *L*_CHOKE_ = 10 mH, was used to stabilize the output current of the TX circuits. The measured efficiency of the designed PA was 91% at 1 MHz, where *L*_TX_ and *R*_TX_ are the inductance and resistance of the TX coil, respectively. The inductively coupled RX circuit consists of three LC series resonant circuits formed by three RX coils and their resonant capacitors, *C*_RX_. *L*_RX_ and *R*_RX_ are the inductance and resistance of the proposed 3D RX. Three full wave bridge rectifiers built with the Schottky diode DB2S20500L and surface mount package of dimensions 1.6 × 0.8 × 0.6 mm^3^, were connected in a series rectification topology. The three outputs were then DC-combined and shared the same DC filter where *C_D_* = 100 pF, *L*_DC_ = 500 nH [42] and *C*_DC_ = 1 μF with the same value of the load resistor, *R_LDC_*, considered in the PTE optimization.

The CSE of the WPT system was 0.7% with the PA, multi-TX WPT link, and Shottky diode-based bridge rectifier displaying respective efficiencies of 91%, 1%, and 75%. For a total input power of 10 W, i.e., input of 5 W for unit TX circuit, an output power of 70 mW is achieved which is higher than current commercial CE [43,44]. The proposed system can supply approximately 50 mW of power inside the phantom in the worst cases of angular and translational misalignment. It is possible to increase the output power of the proposed system by improving the efficiency of the class-E amplifier. In research of Liu (et al.) [41], the propose class-E amplifier can achieve maximum efficiency of 99.3% for an input signal duty ratio of 0.097. This can lead to an output power of 75 mW for the proposed WPT system in this paper. It should be possible to improve the CSE to 0.8% by utilizing high efficiency (more than 87%) rectifier circuits based application specific integrated chip (ASIC) [45,46]. Furthermore, the utilization of the modern 3D printing technology can lead to a more methodological manufacturing technique for the miniaturized 3D RX coil. Compared to the hand wound RX coil this technique can provide more space for layers and turns in the 3D RX and improve the overall PTE and CSE of the system. 

## 6. Conclusions

In this paper, a 3D RX coil and multi-TX-based WPT system are presented for CE application. The WPT system operated at 1 MHz demonstrates a CSE of 0.7% at 10 cm separation distance inside the phantom. The proposed 8.9 mm diameter and 4.8 mm thick RX coil can easily fit into a standard endoscopic capsule. The measured results demonstrate promising PTE at larger separation distance and complete capsule rotation inside phantom. Implementing the TX coils using flexible substrate facilitates easy mounting of the transmitter and portability. Tissue safety is verified by simulating SAR and measuring temperature variations inside a tissue phantom. The proposed 3D WPT system is shown, therefore, to be a potential candidate as an alternative power source for CE. 

## Figures and Tables

**Table 1:** Optimized transmitter (TX) and receiver (RX) coil parameters.

Parameter	*R* (mm)	*w* (mm)	*s* (mm)	*N*	*L_F_* (μH)	*Q*
TX	101	2.5	2	14	30	88
RX (*x*-, *y-* and *z*-axis)	4.45	0.2	-	35	8.3	33.5

**Table 2:** Performance comparison.

Parameter	[20]	[38]	[39]	[37]	This Work
TX Diameter (mm)	400	220	23.5	200	200
RX size (mm^2^)	11.5 × 11.5	9 × 6	15 × 7	8 × 8	8.9 × 4.8
Axes (D)	3	1	1	3	3
Distance (cm)	20	7	5	10	10
WL-PTE (%)	5.02	0.7	1.21	1.3	1
*f* (MHz)	0.218	16.47	433.9	5	1
SAR (W/kg)	8	1.74	2.54	0.86	0.66
Test environment	Air	Pig muscle	Duck intestine	Muscle phantom	Muscle phantom
**FOM (×10^−3^) (25)**	**4.08**	**0.0824**	**0.009**	**8.23**	**80**

WL-PTE: Wireless link power transfer efficiency.

SAR: Specific absorption rate.

FOM: Figure of merit.
